# Supplementary material for: Branched Sulfonimide-Based Proton Exchange Polymer Membranes from Poly(Phenylenebenzopheneone)s for Fuel Cell Applications
Source: Membranes (Basel). 2021 Feb 27;11(3):168. doi: 10.3390/membranes11030168 (PMC7997320; doi:10.3390/membranes11030168)
Supplement: Supplementary file 1 [file membranes-11-00168-s001.pdf]

membranes

Supplementary Materials

Branched Sulfonyl Imide-Based Proton Exchange Polymer Membranes from poly(phenylenebenzopheneone)s for Fuel Cells Application

Sabuj Chandra Sutradhar [1,†], Sujin Yoon [1,†], Taewook Ryu [1], Lei Jin [1], Wei Zhang [1], Whangi Kim [1] and Hohyoun Jang [2*]

[1] Department of Applied Chemistry, Konkuk University, Chungju 27478, Korea; sabujchandra@gmail.com (S.C.S.); ysj920126@naver.com (S.Y.); gundam0924@naver.com (T.R.); jinlei8761@naver.com (L.J.); arno_zw@hotmail.com (W.Z.); wgkim@kku.ac.kr (W.K.)
[2] Department of Liberal Art, Konkuk University, Chungju 27478, Korea
* Correspondence: 200417450@kku.ac.kr; +82-43-840-4764 (ext. 410)
† These authors contributed equally to this work.

Scheme

Scheme S1. Synthesis route for PBP monomer.

Figures

Figure S1. ^{1}H NMR of PBP monomer.

Scheme

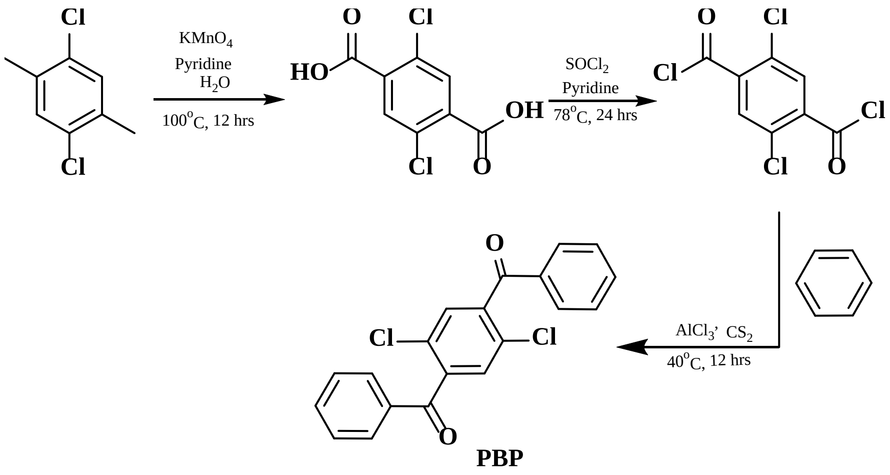

Scheme S1. Synthesis route for PBP monomer.

Figures

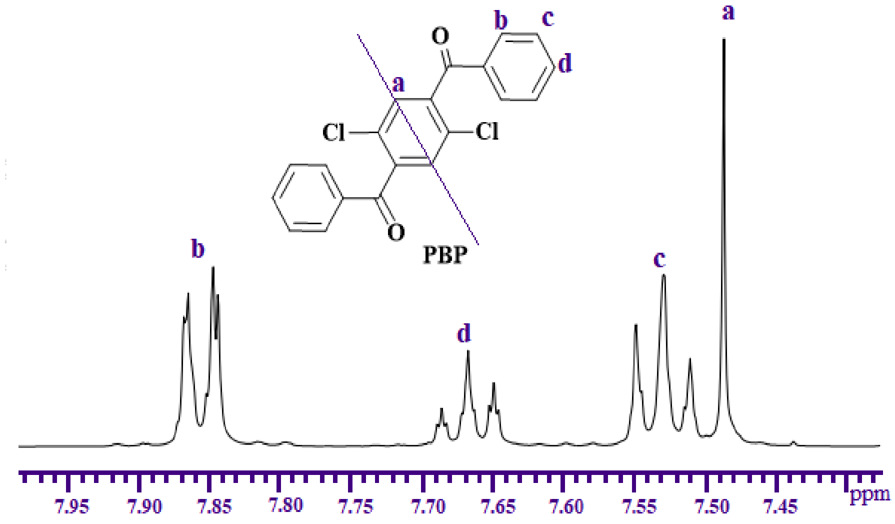

Figure S1. ^{1}H NMR of PBP monomer.
